# A Chromosome-Scale Genome Assembly of *Mitragyna speciosa* (Kratom) and the Assessment of Its Genetic Diversity in Thailand

**DOI:** 10.3390/biology11101492

**Published:** 2022-10-12

**Authors:** Wirulda Pootakham, Thippawan Yoocha, Nukoon Jomchai, Wasitthee Kongkachana, Chaiwat Naktang, Chutima Sonthirod, Srimek Chowpongpang, Panyavut Aumpuchin, Sithichoke Tangphatsornruang

**Affiliations:** 1 National Omics Center, National Science and Technology Development Agency (NSTDA), Pathum Thani 12120, Thailand; 2 National Biobank of Thailand, National Science and Technology Development Agency (NSTDA), Pathum Thani 12120, Thailand

**Keywords:** *Mitragyna*, chromosome-scale genome assembly, PacBio, Hi-C, Iso-seq, genetic diversity

## Abstract

**Simple Summary:**

*Mitragyna speciosa* (Kratom) is a narcotic plant indigenous to Southeast Asian countries, including Thailand. Traditionally, *M. speciosa* has been used as medicine to treat diarrhea and has anti-coughing, analgesic, and fever-reducing properties. Its leaves are commonly chewed by workers during physical labor for their coca-like stimulant effect to increase stamina and endurance. To identify important bioactive alkaloids with potential pharmaceutical uses, we performed a whole genome sequencing of Kratom to obtain information relating to the gene content in its genome, which will facilitate an improved understanding of the biosynthesis pathway and provide resources for assessing the genetic diversity in *M. speciosa.*

**Abstract:**

*Mitragyna speciosa* (Kratom) is a tropical narcotic plant native to Southeast Asia with unique pharmacological properties. Here, we report the first chromosome-scale assembly of the *M. speciosa* genome. We employed PacBio sequencing to obtain a preliminary assembly, which was subsequently scaffolded using the chromatin contact mapping technique (Hi-C) into 22 pseudomolecules. The final assembly was 692 Mb with a scaffold N50 of 26 Mb. We annotated a total of 39,708 protein-coding genes, and our gene predictions recovered 98.4% of the highly conserved orthologs based on the BUSCO analysis. The phylogenetic analysis revealed that *M. speciosa* diverged from the last common ancestors of *Coffea arabica* and *Coffea canephora* approximately 47.6 million years ago. Our analysis of the sequence divergence at fourfold-degenerate sites from orthologous gene pairs provided evidence supporting a genome-wide duplication in *M. speciosa*, agreeing with the report that members of the genus *Mitragyna* are tetraploid. The STRUCTURE and principal component analyses demonstrated that the 85 *M. speciosa* accessions included in this study were an admixture of two subpopulations. The availability of our high-quality chromosome-level genome assembly and the transcriptomic resources will be useful for future studies on the alkaloid biosynthesis pathway, as well as comparative phylogenetic studies in *Mitragyna* and related species.

## 1. Introduction

*Mitragyna speciosa* (also known as Kratom in Thailand) is a tropical evergreen indigenous to Southeast Asia and is commonly grown in Thailand, Malaysia, Vietnam, and Papua New Guinea islands [1,2]. *Mitragyna* is a small genus belonging to the family Rubiaceae. Four species of *Mitragyna* (*Mitragyna diversifolia*, *Mitragyna hirsuta*, *Mitragyna rotundifolia*, and *M. speciosa*) are widely distributed in Thailand, especially from the central to southern parts of the country. Among these four, *M. speciosa* is the only species that is considered as a narcotic plant with specific medicinal importance [1]. Traditionally, *M. speciosa* has been used as medicine to treat diarrhea and has anti-coughing, analgesic, and fever-reducing properties. Its leaves are commonly chewed by workers during physical labor for their coca-like stimulant effect to increase stamina and endurance. In addition, *M. speciosa* has a medical use for treating chronic pain, and it is effective in relieving opioid-related withdrawal symptoms for heroin or morphine addicts [3,4].

Kiehn (1995) reported that *Mitragyna* species are tetraploid with a chromosome number of 2*n* = 4*x* = 44 [5]. Chromosome staining with DAPI was also carried out to confirm the chromosome number of 44 in the mitotic metaphase [6]. However, the identity of progenitor species that underwent hybridization to give rise to *M. speciosa* is currently unknown. The majority of published scientific literature on *M. speciosa* focuses mainly on its chemical composition with mitragynine, the principal compound responsible for analgesic activity, being the most studied alkaloid from this psychoactive medicinal plant [7,8,9,10]. Although mitragynine possesses potent opioid agonist properties, mediating μ- and κ-opioid receptors, its chemical structure is different from those of morphine and other narcotic painkillers [11]. In addition to mitragynine, 54 known alkaloids have successfully been isolated and identified in this species. Due to interest in a diverse range of bioactive alkaloids produced by *M. speciosa*, the draft genome sequence has recently been assembled using the linked-read 10× Genomics technology [6]. The reported assembly encompasses 1.12 Gb and contains 17,031 scaffolds with an N50 scaffold size of 1.02 Mb. To improve the quality and the contiguity of the genome assembly, we employed the Pacific Biosciences (PacBio) long-read single molecule real-time sequencing to generate the preliminary assembly of *M. speciosa*, which was subsequently scaffolded using the chromatin contact mapping (Hi-C) technology to achieve a final assembly that contains 22 pseudomolecules, corresponding to the diploid chromosome number in *M. speciosa*. Our high-quality, chromosome-level assembly of the *M. speciosa* genome provides a valuable resource for identifying bioactive alkaloids with potential pharmaceutical uses and for studying Kratom genetic diversity, population structure, and comparative genomics with related species.

## 2. Materials and Methods

### 2.1. Genome Size Estimation

To estimate the nuclear DNA content using flow cytometry, fresh leaf tissues were cut into small pieces with a sharp razor blade and analyzed using the protocol in [12]. We used the Galbraith’s buffer reported in [13] as a nuclear isolation buffer. Nuclei were stained with 50 ug/mL of propidium iodide (Thermo Fisher Scientific, Waltham, MA, USA). Tomato (*Solanum lycopersicum*) was used as the internal DNA reference standard.

### 2.2. Plant Materials and DNA/RNA Isolation

**Figure 1:**
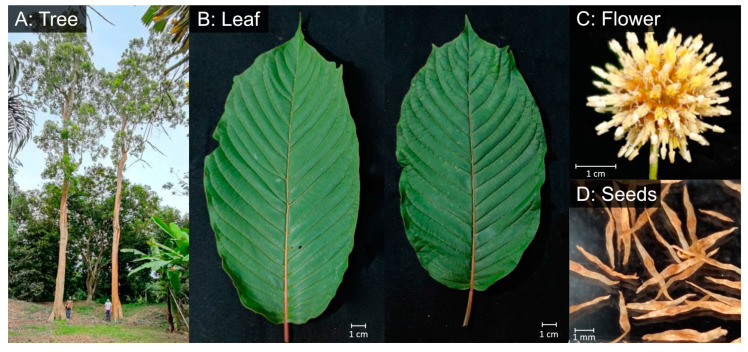
Morphology of *M. speciosa.* (**A**) Tree, (**B**) leaves, (**C**) flower, and (**D**) seeds.

For high-molecular-weight DNA extraction (whole genome sequencing), healthy leaf tissues were collected from a mature *M. speciosa* tree (Figure 1; Sample name ‘RV’ in Supplementary Table S1), immediately frozen, and stored in liquid nitrogen until use. DNA was isolated following the protocol in [14]. Briefly, the frozen tissue was ground in liquid nitrogen and CTAB buffer was added. DNA was extracted from the aqueous phase using 25:24:1 phenol:chloroform:isoamyl alcohol and precipitated in 100% ethanol. DNA pellets were washed twice with 70% ethanol, air-dried, and resuspended in 10 mM Tris-HCl pH 8.0. After purification with the Ampure PB beads (Pacific Biosciences, Menlo Park, CA, USA), DNA integrity was evaluated using Pippin Pulse Electrophoresis System (Sage Science, Beverly, MA, USA). For the genetic diversity study, we collected leaf tissues from a total of 85 *M. speciosa* accessions from 15 provinces in Thailand (Supplementary Figure S1 and Table S1). DNA was isolated using the Qiagen DNeasy Plant Mini Kit following the manufacturer’s protocol (Qiagen, Hilden, Germany). For transcriptome sequencing, leaf, stem, and flower tissues were collected from the same individual used for whole genome sequencing. Total RNA was extracted using the CTAB buffer and 25:24:1 phenol:chloroform:isoamyl alcohol and precipitated overnight in ¼ vol 8M LiCl. RNA pellets were washed twice with 70% ethanol, air-dried, and resuspended in RNase-free water.

### 2.3. Genome and Isoform Sequencing (Iso-seq) Library Preparation

For whole genome sequencing, a SMRTbell library with an insert size of 12,000 nt was prepared from the high molecular weight DNA template using SMRTbell Express Template Prep Kit 2.0 and sequenced on the PacBio Sequel system (Pacific Biosciences, Menlo Park, CA, USA). Sequencing was performed with the Sequel Binding Kit 2.0 using a 20-h movie collection time following the manufacturer’s protocol (Pacific Biosciences, Menlo Park, CA, USA). For transcriptome sequencing, Iso-seq libraries were prepared using the NEBNext Single Cell/Low Input cDNA Synthesis and Amplification Module (New England Biolabs, Ipswich, MA, USA), Iso-Seq Express Oligo Kit, and SMRTbell Express Template Prep Kit 2.0 (Pacific Biosciences, Menlo Park, CA, USA). Iso-seq was performed as described above.

### 2.4. Hi-C Library Preparation and Sequencing

A chromosome conformation capturing technique (Hi-C) was conducted by Biomarker Technologies (Beijing, China) to scaffold the preliminary assembly into a chromosome-level assembly. For Hi-C library preparation, the *M. speciosa* sample was fixed with formaldehyde, and fixed DNA was digested with *Hind*III restriction endonuclease. The overhanging ends were filled with biotinylated nucleotides, and DNA fragments were circularized by blunt-end ligation. After the cross-linking was reversed, DNA was purified and sheared into fragments of 300–700 bp. Biotinylated fragments were captured with streptavidin beads. Purified fragments were used to construct a sequencing library, which was subsequently sequenced on the Illumina platform (PE150; Illumina, San Diego, CA, USA) to produce 398,879,554 read pairs.

### 2.5. PacBio Draft Assembly and Hi-C Scaffolding

A total of 4,049,699 PacBio raw reads totaling 36.8 Gb were subjected to read correction, trimming, overlap detection and de novo assembly by Canu v1.9 [15] using the following parameters: genomeSize = 700 m, correctedErrorRate = 0.040. For other parameters, default settings were used. An estimated genome size of 700 Mb was assumed according to our flow cytometry results (see the ‘Results’ section below). The polishing was carried out using the GenomicConcensus package in the SMRT Link v6.0 (https://github.com/PacificBiosciences/GenomicConsensus, accessed on 6 September 2022). The PacBio draft assembly (used as an input for the Hi-C scaffolding) was cut into 50-kb fragments and reassembled based on Hi-C data. Contigs in the preliminary assembly that could not be recovered in the corrected assembly were regarded as potentially misassembled regions (in those regions, positions with low Hi-C coverage were defined as error sites). The corrected contigs were further scaffolded into a chromosome-level assembly by LACHESIS [16] with the following parameter setting: CLUSTER_MIN_RE_SITES = 21; CLUSTER_MAX_LINK_DENSITY = 2; ORDER_MIN_N_RES_IN_TRUNK = 15; ORDER_MIN_N_RES_IN_SHREDS = 15. After this step, placement and orientation errors exhibiting obvious discrete chromatin interaction patterns were manually adjusted. After the manual adjustment, a total of 672 Mb sequences were anchored onto 22 chromosomes.

### 2.6. Genome Assembly Evaluation

The quality of the final genome assembly was assessed by aligning short-read DNA sequences (RADseq data) and transcriptome (Iso-seq) data from this study using BWA version 0.7.17-r1188 for DNA sequence alignment and HISAT2 version 2.2.0 for RNA alignment. We also tested for the presence and completeness of the orthologs using the Benchmarking Universal Single-Copy Orthologues (BUSCO) version 4.0.5 [17] and the Embryophyta OrthoDB release 10 [18]. 

To identify repetitive element families in the genome assembly, RepeatModeler version 2.0.3 (http://www.repeatmasker.org/RepeatModeler/, last accessed on 16 August 2022) was used to construct a de novo repeat library. This pipeline employed two distinct repeat discovery algorithms, RECON (version 1.08) and RepeatScout (version 1.0.6), to identify the boundaries of repetitive elements and build consensus models of interspersed repeats [19,20]. We aligned repeat sequences in the library to GenBank’s nr protein database using BLASTX (e-value cutoff = 10^−6^) to ensure that they did not contain large families of protein-coding sequences.

To identify protein-coding sequences in the unmasked assembly, we used EvidenceModeler (EVM) version 1.1.1 to combine evidences from RNA-based predictions, homology-based predictions, and ab initio predictions [21]. For RNA-based prediction, we used evidence from PacBio Iso-seq data obtained from leaf, stem, and flower tissues. Full-length transcripts were mapped to the final assembly using the genomic mapping and alignment program (GMAP) version 2020-09-12 [22]. Protein sequences from *Coffea arabica*, *Coffea canephora*, *S. lycopersicum*, *M. speciosa*, and *Arabidopsis thaliana* available on the public databases were aligned to the unmasked genome using AAT [23]. Protein-coding gene predictions were obtained with Augustus version 3.2.1 [24] trained with *C. arabica*, *C. canephora*, *S. lycopersicum*, and *M. speciosa* PASA transcriptome alignment assembly using *M. speciosa* alignment files as inputs. All gene predictions were integrated by EVM to generate consensus gene models using the following weights for each evidence type: PASA2—1, GMAP—0.5, AAT—0.3 and Augustus—0.3. Any predicted genes that had more than 20% overlapping sequence with repetitive sequences or had no RNA-seq support were excluded from the list of annotated genes.

### 2.7. Phylogenetic Analyses and Comparative Genomics

OrthoFinder version 2.4.0 [25] was used to identify orthologous groups in *M. speciosa*, *Amborella trichopoda*, *A. thaliana*, *C. arabica*, *C. canephora*, *Erythranthe guttata*, *Populus trichocarpa*, *Prunus persica*, *Ricinus communis*, *S. lycopersicum*, *Theobroma cacao*, and *Vitis vinifera*. We constructed a phylogenetic tree based on protein sequences from single-copy orthologous groups using RAxML-NG program version 1.0.2 [26]. We first aligned protein sequences in each single-copy orthologous group with MUSCLE [27] and removed alignment gaps with trimAI version 1.4 rev15 [28] using the automated1 heuristic method. We subsequently concatenated alignment blocks using catsequences program (https://github.com/ChrisCreevey/catsequences, last accessed on 22 August 2022), and the substitution model for each block was estimated using the ModelTest-NG program version 0.1.7 [29]. The outputs were used to compute a maximum-likelihood phylogenetic tree. Divergence times were estimated using the MCMCtree software version 4.0 (PAML 4 package) [30] using the relaxed-clock model with the known divergence time between *P. trichocarpa* and *R. communis*, which was estimated at 105–120 million years ago (MYA) [31,32].

### 2.8. Genome Synteny Analysis

McscanX [33] was used to analyze the collinearity within the *M. speciosa* genome and between *M. speciosa–C. arabica* and *M. speciosa–C. robusta* genomes. *M. speciosa* amino acid sequences were aligned against themselves, *C. arabica*, or *C. canephora* using BLASTP (with an e-value cutoff of 10^−10^) in order to identify putative paralogs. Intragenic homeologous blocks were defined as regions of ten or more genes with colinear or nearly colinear runs of paralogs elsewhere in the genome with fewer than six intervening genes. These intragenic homeologous blocks were visualized using CIRCOS version 0.69.8 [34]. Similarly, we also performed pairwise comparisons of input protein sequences from *M. speciosa*, *C. canephora*, and *C. arabica*. Clustering was carried out using OrthoMCL software version 2.0.9 [35] based on a Markov clustering algorithm (MCL). Syntenic blocks between *M. speciosa*, *C. canephora*, and *C. arabica* were identified by MCscanX and plotted with CIRCOS using the criteria mentioned above (at least ten colinear genes and fewer than six intervening genes allowed).

### 2.9. Population Structure and Genetic Diversity Analyses

Short-read data (RAD-seq) from 85 *M. speciosa* accessions collected in Thailand were used to analyze the population structure. We first mapped the sequencing reads to the final genome assembly using BWA v0.7.17, and single nucleotide polymorphism (SNP) markers were identified using GATK HaplotypeCaller 3.8 [36]. A set of 2492 SNP markers at fourfold-degenerate sites with the following criteria were used for the analyses: (I) depth coverage between 20× and 200×, (II) minor allele frequency > 0.05, and (III) less than 10% missing data. A maximum-likelihood tree with 1000 bootstrap replicates was constructed using the analysis of phylogenetic and evolution (APE) software version 5.5 in the R package [37]. To evaluate the population structure, we employed the STRUCTURE program version 2.3.4 [38] using the same set of SNP markers (for the phylogenetic tree) and 10,000 iterations with the number of clusters (*K*) of 2–4. The level of genetic diversity in the population investigated was estimated by calculating the gene diversity (GD, or expected heterozygosity), polymorphism information content (PIC), observed heterozygosity (*H_O_*), and minor allele frequency (MAF) using the PowerMarker software version 3.25 [39]. Principal component analysis (PCA) was performed based on genetic distances (2492 SNP markers) among 85 *M. speciosa* accessions computed with TASSEL version 5.2.77 [40]. Principal components were generated using the covariance method, and eigenvalues were generated to determine the proportion of variation explained by each principal component. The first and second principal components were plotted using R software package ggplot2 version 3.3.4 [41]. 

## 3. Results

### 3.1. M. speciosa Genome Assembly and Annotation

**Figure 2:**
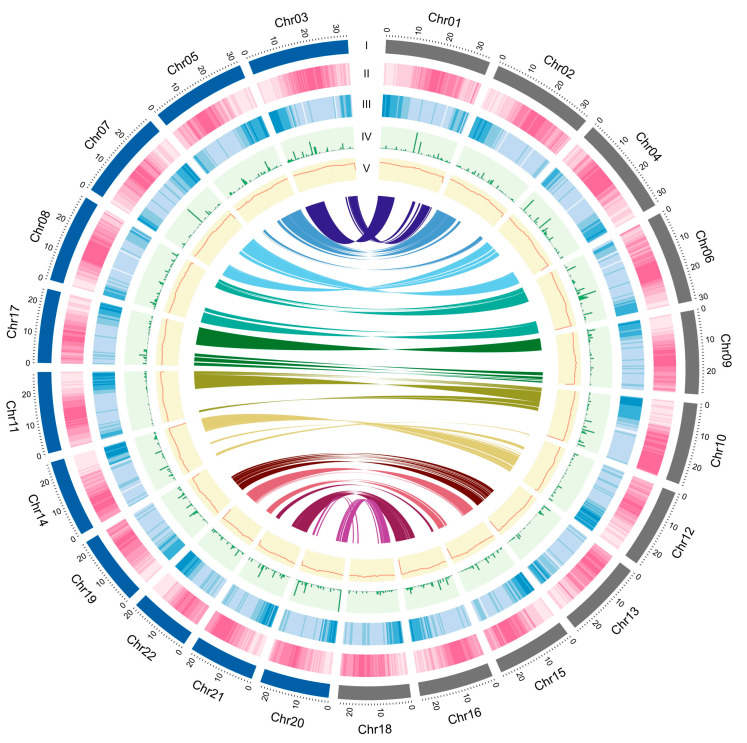
Genomic landscape of *M. speciosa*. (I) A physical map of 22 pseudomolecules (chromosomes) numbered according to size (in Mb); (II) Content of repetitive sequences shown as proportion of genomic regions covered by repetitive sequences in 250-kb windows; (III) Gene density represented by the number of genes in 250-kb windows; (IV) SNP density represented by number of SNP markers in 250-kb windows; (V) GC content represented by the percentage of G + C bases in 250-kb windows. Syntenic blocks in the genome are displayed by colored lines.

To obtain a preliminary draft assembly of *M. speciosa*, whole-genome shotgun sequencing was carried out using PacBio long-read technology, and 36.8 Gb of sequencing data (4,049,699 reads) were generated. Based on the estimated genome size of 727 Mb obtained from the DNA flow cytometry (Supplementary Figure S2), these raw sequencing data represented ~54× coverage of the genome size. A preliminary PacBio assembly yielded a draft genome of 692 Mb in 4259 scaffolds with an N50 length of 0.9 Mb (Table 1). This draft assembly was subsequently scaffolded using the long-range chromatin fixation technique (Hi-C) into a chromosome-level assembly containing 22 pseudomolecules > 10 Mb in length (from here on, they will be referred to as chromosomes; Figure 2, Supplementary Figure S3). The genus Mitragyna has been reported as a tetraploid with a base chromosome number of 11 (2*n* = 4*x* = 44) [5,6]. The 22 chromosomes corresponded to the diploid chromosome number and covered 602 Mb, or 87.04% of the 692-Mb assembly.

To evaluate the quality of our assembly, short-read genomic DNA data (from RAD-seq) were aligned to the genome sequence, and 99.62% of the reads could be mapped to the *M. speciosa* genome. In addition, Iso-seq reads were aligned to the assembly, and 99.72% of the transcripts were mapped to the genome. An additional assessment of the gene space completeness was carried out using the BUSCO software and a plant-specific database of 1440 genes [17]. Predicted gene models in our *M. speciosa* assembly covered as much as 98.4% of the highly conserved orthologs in the Embryophyta lineage (81.6% classified as ‘complete and single-copy’, 15.4% as ‘complete and duplicated’, 1.4% as ‘fragmented’; Table 1). Results from the alignment and BUSCO analysis suggested that our chromosome-scale *M. speciosa* assembly is of high quality.

To annotate the genome assembly, three approaches were employed: ab initio prediction, homology-based search, and transcript-based evidence. A total of 42,873 predicted gene models and 39,708 protein-coding genes (92.6% of predicted gene models; Supplementary Table S2) were identified. We were able to assign gene ontology (GO) to 35,485 protein-coding genes (82.7% of predicted gene models; Supplementary Figure S4). The most prevalent GO terms associated with cellular components were the integral components of membrane, nucleus, and cytoplasm, while the largest categories of genes associated with molecular function and biological processes were ATP binding and protein phosphorylation, respectively. In addition, 70.3, 38.2 and 26.2% of the predicted gene models could be functionally annotated using the Swissprot, EC, and KEGG databases, respectively (Supplementary Table S3). The average GC content in the *M. speciosa* genome was 34.59% (Table 1), which was close to the average GC content in introns (33.8%; Supplementary Table S2), whereas the average GC content in exons was much higher at 43.3% (Supplementary Table S2).

We identified a number of genes involved in the specialized metabolic pathways that lead to the production of strictosidine, the intermediate in the monoterpene indole alkaloid biosynthesis (Supplementary Figure S5). Several gene families contain multiple homoeologs including those encoding key enzymes, such as loganic acid methyltransferase (LAMT), secologanin synthase (SLS), and strictosidine synthase (STR). The presence of duplicated genes is likely the consequence of the tetraploidization event. Interestingly, some of the genes encoding enzymes in the methylerythriol phosphate (MEP) pathway are present in single copies (Supplementary Figure S5), suggesting that duplicated genes may have been lost or degraded into non-functional genes after the polyploidization [42].

### 3.2. Identification of Repetitive Elements in the M. speciosa Genome

The *M. speciosa* genome assembly contained a total of 354.61 Mb of repetitive elements, representing 51.21% of the assembly (Table 2). A genome-wide distribution plot showed that DNA transposons and retrotransposons were located in proximity to the centromeric regions (Figure 2). Retrotransposons (122.38 Mb) represented the majority of the repetitive elements identified, comprising 17.83% of the assembly and 34.51% of the total repeated content. The most abundant retrotransposon types were the long terminal repeat (LTR) Copia and Gypsy, occupying 6.36% and 8.48% of the genome assembly.

### 3.3. Comparative Genomics and Phylogenetic Analyses

**Figure 3:**
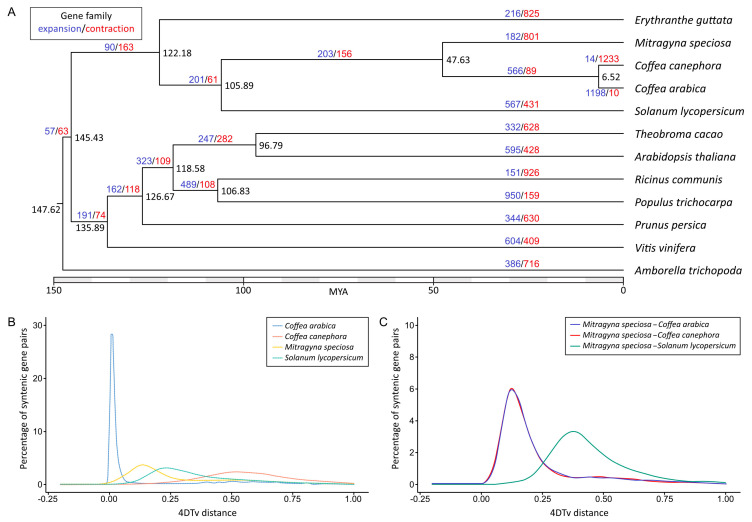
Comparative genomics of *M. speciosa* and related species. (**A**) A maximum-likelihood tree of *M. speciosa*, two *Coffea* species, and nine other plant species constructed based on single-copy orthologous protein sequences. Numbers at each node represent the estimated divergence time in million years ago (MYA). Numbers in blue and red represent the numbers of gene families that have expanded or contracted, respectively, relative to their ancestors. (**B**) The distribution of fourfold synonymous third-codon transversion position (4DTv) distances between paralogous genes in *M. speciosa*, *C. arabica*, *C. canephora*, and *S. lycopersicum*. (**C**) The distribution of 4DTv distances between orthologous genes in *M. speciosa* and *C. arabica*, *C. canephora* and *S. lycopersicum*. Peaks of intraspecific and interspecific 4DTv distributions indicate whole-genome duplication and speciation events, respectively.

To perform comparative genomics analyses, we clustered a total of 452,592 proteins (out of 469,904 input proteins from 12 species) into 28,228 orthologous groups. We then constructed a maximum-likelihood phylogenetic tree using the sequence information from single-copy orthologous genes and calculated the divergence time based on the topology and the branch length. *M. speciosa* diverged from the last common ancestors of *C. arabica* and *C. canephora* approximately 47.6 MYA (Figure 3A), and the last common ancestor of *M. speciosa*, *C. arabica*, and *C. canephora* diverged from *S. lycopersicum* ~106 MYA.

We determined the accumulated sequence divergence at fourfold synonymous third-codon transversion rate (4DTv) between the paralogous gene pairs in *M. speciosa*, *C. Arabica*, *C. canephora*, and *S. lycopersicum* to estimate their relative ages (Figure 3B). After correcting for multiple substitutions, we observed a sharp peak in 4DTv value at 0.012 synonymous transversions per site in *C. arabica*. This corresponded to the period between 1.08 and 0.54 million years ago, during which the hybridization of two progenitor species (*C. canephora* and *Coffea eugenioides*) led to the emergence of *C. arabica* [43]. Comparisons between the 12,198 pairs of paralogous genes residing within 418 duplicated colinear blocks within the *M. speciosa* genome revealed a noticeable peak at 0.139, suggesting that *M. speciosa* has undergone a recent whole-genome duplication (Figure 3B). This was also evidenced by the presence of intragenomic syntenic blocks throughout the genome (Figure 2) and the syntenic blocks between *M. speciosa* and the tetraploid *C. arabica* (Supplementary Figure S6). Interestingly, this peak overlapped with the 4DTv distance of *M. speciosa–C. arabica* and *M. speciosa–C. canephora*, suggesting that the genome-wide duplication event in *M. speciosa* took place in the time frame during which *M. speciosa* diverged from the last common ancestor of the two *Coffea* species (Figure 3C).

### 3.4. Genetic Diversity and Population Structure in M. speciosa

**Figure 4:**
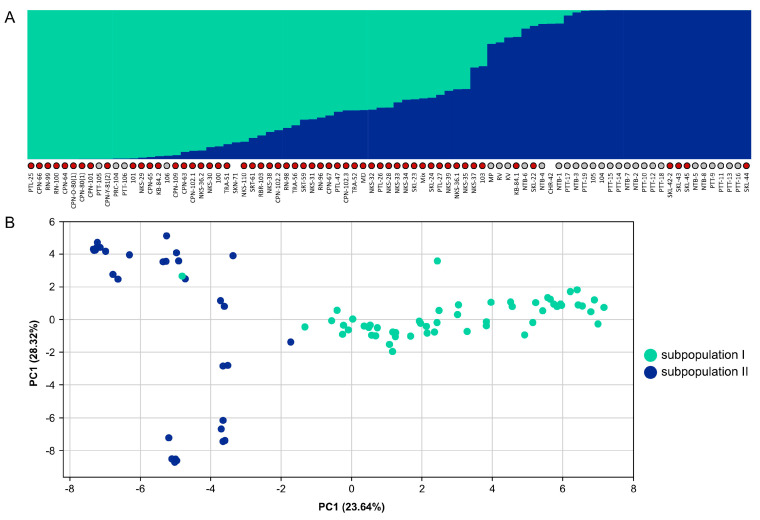
Population structure and genetic diversity of *M. speciosa.* (**A**) Population structure of 85 *M. speciosa* accessions. Each accession is represented by a vertical bar, and the length of each color reflects the proportion contributed by ancestral populations. Accessions collected in Western and Southern Thailand are indicated by red dots while those collected in Central Thailand are indicated by grey dots. (**B**) Principal component analysis (PCA) of 85 *M. speciosa* accessions based on 2492 SNP markers. Each accession is represented by a single dot with colors indicating subpopulations.

We collected and sequenced 85 *M. speciosa* accessions from different regions in Thailand in order to evaluate their genetic diversity and population structure. A total of 2492 SNP loci at fourfold-degenerate sites were used to assess the population structure using the Bayesian model-based clustering approach [44]. We selected the best K value based on the ΔK parameter proposed by [45], and K = 2 seemed to be the best fit for our data (Supplementary Figure S7). The STRUCTURE and PCA analyses demonstrated that the 85 *M. speciosa* accessions sampled in this work represented an admixture of two subpopulations (Figure 4) that appeared to originate from different geographical locations. One subpopulation consisted primarily of individuals collected from Western and Southern Thailand, whereas the other comprised individuals collected from Central Thailand (Supplementary Table S1).

**Figure 5:**
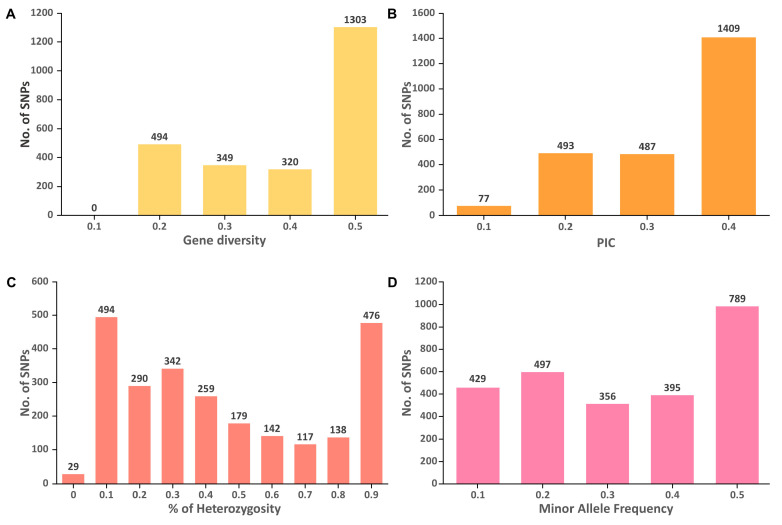
Distribution of (**A**) gene diversity (GD), (**B**) polymorphism information content (PIC), (**C**) percentage of heterozygosity, and (**D**) minor allele frequency for 2492 SNP markers among the 85 *M. speciosa* accessions.

The genetic diversity analysis of 85 *M. speciosa* accessions revealed that the GD ranged from 0.1 to 0.5 with an average of 0.36, while the PIC varied from 0.09 to 0.38 with an average of 0.28 (Figure 5). For the majority of SNPs analyzed, the GD and PIC values were ~0.5 and ~0.4, respectively. The H_O_ values varied from 0.06 to 1 with most of the SNPs possessing H_O_ values of 0.1. The mean values for H_O_ and MAF were 0.5 and 0.28, respectively.

## 4. Discussion

*M. speciosa*, or Kratom, is a tropical plant indigenous to Southeast Asia that produces multiple biologically active phytochemicals with unique pharmacological properties. Here, we present a de novo assembly of the *M. speciosa* genome using the combination of PacBio long-read SMRT technology and the long-range proximity ligation technique (Hi-C). The preliminary assembly obtained from the PacBio sequencing was 692 Mb, slightly smaller than our estimated genome size of 727 Mb (by DNA flow cytometry) and a previously reported genome size of 780 Mb [6]. We subsequently employed the Hi-C method to identify chromosomal interactions using chromosome conformation capture. The Hi-C data provided long-range linkage information of up to tens of megabases that could be used to generate chromosome-level scaffolds. Our final assembly contained 22 pseudomolecules covering 602 Mb or ~87% of the 692-Mb assembly. The genus *Mitragyna* has been reported as a tetraploid with a base chromosome number of 11 (2*n* = 4*x* = 44) [5,6]. We believed that *M. speciosa* is an allotetraploid deriving from the hybridization of two currently unknown progenitors since our Hi-C assembly was phased into 22 chromosomes instead of the haploid chromosome number. We evaluated the representation of highly conserved orthologs using the BUSCO tool and demonstrated that our gene prediction recovered as much as 98.4% of the conserved orthologs in the Embryophyta lineage, suggesting that our genome assembly is more complete than the previously reported assembly, which covered only 88.5% of the BUSCO orthologs [6]. In addition, the contiguity of our chromosome-level assembly (N50 = 26 Mb and contains 2888 scaffolds) is also superior to that of the published assembly (N50 = 1 Mb and contains 17,031 scaffolds) [6]. We also noticed a huge discrepancy between the sizes of our assembly (692 Mb) and the previously published one (1122 Mb) [6]. The dot plot alignment of scaffolds/contigs from these two assemblies revealed several duplicated regions in the previously published assembly (examples shown in Supplementary Figure S8), suggesting that there were residual haplotigs remaining in the previous assembly. Those uncollapsed contigs provided the most likely explanation as to why the previous assembly was significantly larger than both the one presented here and the estimated genome size by DNA flow cytometry.

The analysis of intragenomic synteny and the 4DTv analysis of the paralogous gene pairs in *M. speciosa* revealed the evidence of whole-genome duplication. There appeared to be a one-to-one synteny between 11 pairs of homeologous chromosomes (Figure 1). Interestingly, our 4DTv analyses suggested that the genome-wide duplication in *M. speciosa* occurred during the same period in which *M. speciosa* diverged from the last common ancestor of those two *Coffea* species (Figure 3B). Our evaluation of the *M. speciosa* population structure showed that the 85 individuals sampled in this work could be grouped into two subpopulations. The clustering appeared to be associated with the geographical regions from which the samples were collected, with individuals from Western and Southern Thailand in one subpopulation and those from Central Thailand in another. We also assessed the level of genetic diversity of the population studied and found that among the 85 *M. speciosa* accessions, most of the SNP markers were moderately informative with an average PIC value of 0.28. These molecular markers will be useful for evaluating the genetic diversity of other *M. speciosa* populations and also for future marker-assisted breeding programs.

## 5. Conclusions

With *M. speciosa* emerging as a new cash crop, there has been interest in developing elite cultivars producing bioactive alkaloids with potential pharmaceutical uses. The availability of this high-quality reference genome assembly, the transcriptomic data along with the genomic variation information from the population will enable future studies on gene expression profiling that can improve our understanding of the biosynthetic pathways, studies on phylogeny, and comparative genomics in Rubiaceae family.

## Figures and Tables

**Table 1:** Assembly statistics of *M. speciosa* genome.

	PacBio	PacBio + HiC
N50 scaffold size (bases)	922,929	26,436,849
L50 scaffold number	200	12
N75 scaffold size (bases)	339,531	23,267,248
L75 scaffold number	502	19
N90 scaffold size (bases)	41,110	56,307
L90 scaffold number	1456	159
Assembly size (bases)	692,306,703	692,445,403
Number of scaffolds	4259	2888
Number of scaffolds ≥ 100 kb	862	64
Number of scaffolds ≥ 1 Mb	173	24
Number of scaffolds ≥ 10 Mb	0	22
Longest scaffold (bases)	7,719,426	34,865,628
% N	0	0.02
GC content (%)	34.59	34.59
BUSCO evaluation (% completeness)	-	98.4

**Table 2:** Repeat contents in *M. speciosa* genome.

Types of Repeats	Bases (Mb)	% of the Assembly	% of Total Repeats
DNA transposons	9.98	1.44	3.92
Retrotransposons:			
LINE	17.58	2.54	2.31
SINE	0.001	0.00	0.00
LTR: *Copia*	44.08	6.36	12.93
LTR: *Gypsy*	58.74	8.48	16.96
LTR: Others	1.98	0.28	0.56
Simple sequence repeats	27.71	4.00	7.95
Others	194.54	28.11	55.37
Total	354.61	51.21	

## Data Availability

*M. speciosa* genome assembly and Iso-seq data have been submitted to the DDBJ/EMBL/GenBank databases under Bioproject PRJNA849364 and the following accession numbers: JAMWEH000000000 (genome assembly), SRR20982408 (Iso-seq; leaf), SRR20982407 (Iso-seq; stem) and SRR20982406 (Iso-seq; flower).

## Supplementary Materials

The following supporting information can be downloaded at: https://www.mdpi.com/article/10.3390/biology11101492/s1, Figure S1: A map illustrating the locations of 85 *M. speciosa* accessions in Thailand collected for the population structure study; Figure S2: Genome size estimation by DNA flow cytometry. Histograms of relative DNA contents obtained after analysis of nuclei isolated from leaf tissues of *M. speciosa* and tomato plants (*Solanum lycopersicum*; used as a reference standard); Figure S3: Hi-C interaction matrix maps of *M. speciosa* chromosomes. The contact density is indicated by the color scale on the right from dark red (high density) to white (low density); Figure S4: Gene Ontology (GO) annotation of *M. speciosa* genes in the assembly; Figure S5: Mitragynine biosynthesis pathway and the list of genes in the pathway that were identified in the genome assembly; Figure S6: Synteny between *M. speciosa* and (A) *C. canephora* (B) *C. arabica*; Figure S7: Delta K values for STRUCTURE analysis of 85 *M. speciosa* accessions. Delta K is plotted against the number of modeled gene pools (K); Figure S8: Examples of the dot plot alignment of scaffolds/contigs from our genome assembly (X-axis) and the previously published one (Y-axis). Red circles indicated duplicated regions in the assembly published by Brose et al. (2021). Table S1: List of sequenced *M. speciosa* accessions; Table S2: Annotation statistics for *M. speciosa*; Table S3: Functional annotations of *M. speciosa* protein-coding genes.

## References

[1] Davis A. (2006). Rubiaceae of Thailand—A pictorial guide to indigenous and cultivated genera. Bot. J. Linn. Soc..

[2] Suwanlert S. (1975). A study of kratom eaters in Thailand. Bull. Narc..

[3] Cinosi E., Martinotti G., Simonato P., Singh D., Demetrovics Z., Roman-Urrestarazu A., Bersani F.S., Vicknasingam B., Piazzon G., Li J.-H. (2015). Following “the roots” of Kratom (*Mitragyna speciosa*): The evolution of an enhancer from a traditional use to increase work and productivity in Southeast Asia to a recreational psychoactive drug in western countries. BioMed Res. Int..

[4] Grundmann O. (2017). Patterns of kratom use and health impact in the US—Results from an online survey. Drug Alcohol Depend..

[5] Kiehn M. (1995). Chromosome Survey of the Rubiaceae. Ann. Mo. Bot. Gard..

[6] Brose J., Lau K.H., Dang T.T.T., Hamilton J.P., Martins L.d.V., Hamberger B., Hamberger B., Jiang J., O’Connor S.E., Buell C.R. (2021). The *Mitragyna speciosa* (Kratom) Genome: A resource for data-mining potent pharmaceuticals that impact human health. G3 Genes.

[7] Flores-Bocanegra L., Raja H.A., Graf T.N., Augustinović M., Wallace E.D., Hematian S., Kellogg J.J., Todd D.A., Cech N.B., Oberlies N.H. (2020). The Chemistry of Kratom [*Mitragyna speciosa*]: Updated Characterization Data and Methods to Elucidate Indole and Oxindole Alkaloids. J. Nat. Prod..

[8] Beckett A., Shellard E., Tackie A. (1965). THE MITRAGYNA SPECIES OF ASIA–Part IV. The alkaloids of the leaves of *Mitragyna speciosa* Korth.. Isolation of Mitragynine and Speciofoline1. Planta Med..

[9] Takayama H., Ishikawa H., Kurihara M., Kitajima M., Aimi N., Ponglux D., Koyama F., Matsumoto K., Moriyama T., Yamamoto L.T. (2002). Studies on the Synthesis and Opioid Agonistic Activities of Mitragynine-Related Indole Alkaloids:  Discovery of Opioid Agonists Structurally Different from Other Opioid Ligands. J. Med. Chem..

[10] Karunakaran T., Ngew K.Z., Zailan A.A.D., Mian Jong V.Y., Abu Bakar M.H. (2022). The Chemical and Pharmacological Properties of Mitragynine and Its Diastereomers: An Insight Review. Front. Pharmacol..

[11] Gibbons S., Arunotayanun W., Dargan P.I., Wood D.M. (2013). Chapter 14—Natural Product (Fungal and Herbal) Novel Psychoactive Substances. Novel Psychoactive Substances.

[12] Dolezel J., Bartos J. (2005). Plant DNA flow cytometry and estimation of nuclear genome size. Ann. Bot..

[13] Galbraith D.W., Harkins K.R., Maddox J.M., Ayres N.M., Sharma D.P., Firoozabady E. (1983). Rapid Flow Cytometric Analysis of the Cell Cycle in Intact Plant Tissues. Science.

[14] Pootakham W., Naktang C., Sonthirod C., Kongkachana W., Narong N., Sangsrakru D., Maknual C., Jiumjamrassil D., Chumriang P., Tangphatsornroung S. (2022). Chromosome-level genome assembly of the Indian mangrove (*Ceriops tagal*) revealed a genome-wide duplication event predating the divergence of Rhizophoraceae mangroves. Plant Genome.

[15] Koren S., Walenz B.P., Berlin K., Miller J.R., Bergman N.H., Phillippy A.M. (2017). Canu: Scalable and accurate long-read assembly via adaptive k-mer weighting and repeat separation. Genome Res..

[16] Burton J.N., Adey A., Patwardhan R.P., Qiu R., Kitzman J.O., Shendure J. (2013). Chromosome-scale scaffolding of de novo genome assemblies based on chromatin interactions. Nat. Biotechnol..

[17] Simão F.A., Waterhouse R.M., Ioannidis P., Kriventseva E.V., Zdobnov E.M. (2015). BUSCO: Assessing genome assembly and annotation completeness with single-copy orthologs. Bioinformatics.

[18] Kriventseva E.V., Tegenfeldt F., Petty T.J., Waterhouse R.M., Simao F.A., Pozdnyakov I.A., Ioannidis P., Zdobnov E.M. (2015). OrthoDB v8: Update of the hierarchical catalog of orthologs and the underlying free software. Nucleic Acids Res..

[19] Price A.L., Jones N.C., Pevzner P.A. (2005). De novo identification of repeat families in large genomes. Bioinformatics.

[20] Bao Z., Eddy S.R. (2002). Automated de novo identification of repeat sequence families in sequenced genomes. Genome Res..

[21] Haas B.J., Salzberg S.L., Zhu W., Pertea M., Allen J.E., Orvis J., White O., Buell C.R., Wortman J.R. (2008). Automated eukaryotic gene structure annotation using EVidenceModeler and the program to assemble spliced alignments. Genome Biol..

[22] Wu T., Watanabe C. (2005). GMAP: A genomic mapping and alignment program for mRNA and EST sequences. Bioinformatics.

[23] Huang X., Adams M.D., Zhou H., Kerlavage A.R. (1997). A tool for analyzing and annotating genomic sequences. Genomics.

[24] Stanke M., Steinkamp R., Waack S., Morgenstern B. (2004). AUGUSTUS: A web server for gene finding in eukaryotes. Nucleic Acids Res..

[25] Emms D.M., Kelly S. (2019). OrthoFinder: Phylogenetic orthology inference for comparative genomics. Genome Biol..

[26] Stamatakis A. (2006). RAxML-VI-HPC: Maximum likelihood-based phylogenetic analyses with thousands of taxa and mixed models. Bioinformatics.

[27] Edgar R.C. (2004). MUSCLE: Multiple sequence alignment with high accuracy and high throughput. Nucleic Acids Res..

[28] Capella-Gutiérrez S., Silla-Martínez J.M., Gabaldón T. (2009). trimAl: A tool for automated alignment trimming in large-scale phylogenetic analyses. Bioinformatics.

[29] Darriba D., Posada D., Kozlov A.M., Stamatakis A., Morel B., Flouri T. (2020). ModelTest-NG: A new and scalable tool for the selection of DNA and protein evolutionary models. Mol. Biol. Evol..

[30] Yang Z. (2007). PAML 4: Phylogenetic analysis by maximum likelihood. Mol. Biol. Evol..

[31] Xi Z., Ruhfel B.R., Schaefer H., Amorim A.M., Sugumaran M., Wurdack K.J., Endress P.K., Matthews M.L., Stevens P.F., Mathews S. (2012). Phylogenomics and a posteriori data partitioning resolve the Cretaceous angiosperm radiation Malpighiales. Proc. Natl. Acad. Sci. USA.

[32] Davis C.C., Webb C.O., Wurdack K.J., Jaramillo C.A., Donoghue M.J. (2005). Explosive Radiation of Malpighiales Supports a Mid-Cretaceous Origin of Modern Tropical Rain Forests. Am. Nat..

[33] Wang Y., Tang H., Debarry J.D., Tan X., Li J., Wang X., Lee T.H., Jin H., Marler B., Guo H. (2012). MCScanX: A toolkit for detection and evolutionary analysis of gene synteny and collinearity. Nucleic Acids Res..

[34] Krzywinski M.I., Schein J.E., Birol I., Connors J., Gascoyne R., Horsman D., Jones S.J., Marra M.A. (2009). Circos: An information aesthetic for comparative genomics. Genome Res..

[35] Li L., Stoeckert C.J., Roos D.S. (2003). OrthoMCL: Identification of ortholog groups for eukaryotic genomes. Genome Res..

[36] McKenna A., Hanna M., Banks E., Sivachenko A., Cibulskis K., Kernytsky A., Garimella K., Altshuler D., Gabriel S., Daly M. (2010). The Genome Analysis Toolkit: A MapReduce framework for analyzing next-generation DNA sequencing data. Genome Res..

[37] Paradis E., Claude J., Strimmer K. (2004). APE: Analyses of Phylogenetics and Evolution in R language. Bioinformatics.

[38] Falush D., Stephens M., Pritchard J.K. (2003). Inference of population structure using multilocus genotype data: Linked loci and correlated allele frequencies. Genetics.

[39] Liu K., Muse S.V. (2005). PowerMarker: An integrated analysis environment for genetic marker analysis. Bioinformatics.

[40] Bradbury P.J., Zhang Z., Kroon D.E., Casstevens T.M., Ramdoss Y., Buckler E.S. (2007). TASSEL: Software for association mapping of complex traits in diverse samples. Bioinformatics.

[41] Villanueva R.A.M., Chen Z.J. (2019). ggplot2: Elegant Graphics for Data Analysis (2nd ed.). Meas. Interdiscip. Res. Perspect..

[42] Edger P.P., Pires J.C. (2009). Gene and genome duplications: The impact of dosage-sensitivity on the fate of nuclear genes. Chromosome Res..

[43] Bawin Y., Ruttink T., Staelens A., Haegeman A., Stoffelen P., Mwanga Mwanga J.-C.I., Roldán-Ruiz I., Honnay O., Janssens S.B. (2021). Phylogenomic analysis clarifies the evolutionary origin of *Coffea arabica*. J. Syst. Evol..

[44] Pritchard J.K., Stephens M., Donnelly P. (2000). Inference of population structure using multilocus genotype data. Genetics.

[45] Evanno G., Regnaut S., Goudet J. (2005). Detecting the number of clusters of individuals using the software STRUCTURE: A simulation study. Mol. Ecol..

