# Supplementary material for: A Chromosome-Scale Genome Assembly of Mitragyna speciosa (Kratom) and the Assessment of Its Genetic Diversity in Thailand
Source: Biology (Basel). 2022 Oct 12;11(10):1492. doi: 10.3390/biology11101492 (PMC9598492; doi:10.3390/biology11101492)
Supplement: Supplementary file 1 [file biology-11-01492-s001.zip › Supplementary/REVISED M speciosa Supplementary Figures.pdf]

Supplementary Figures

A chromosome-scale genome assembly of Mitragyna speciosa (Kratom) and the assessment of its genetic diversity in Thailand

Wirulda Pootakham[1], Thippawan Yoocha[1], Nukoon Jomchai[1], Wasitthee Kongkachana[1], Chaiwat Naktang[1], Chutima Sonthirod[1], Srimek Chowpongpang[2], Panyavut Aumpuchin[2], Sithichoke Tangphatsornruang[1,*].

[1]National Omics Center, National Science and Technology Development Agency (NSTDA), Pathum Thani, Thailand.
[2]National Biobank of Thailand, National Science and Technology Development Agency (NSTDA), Pathum Thani, Thailand.

*Corresponding author
Sithichoke Tangphatsornruang (sithichoke.tan@nstda.or.th)

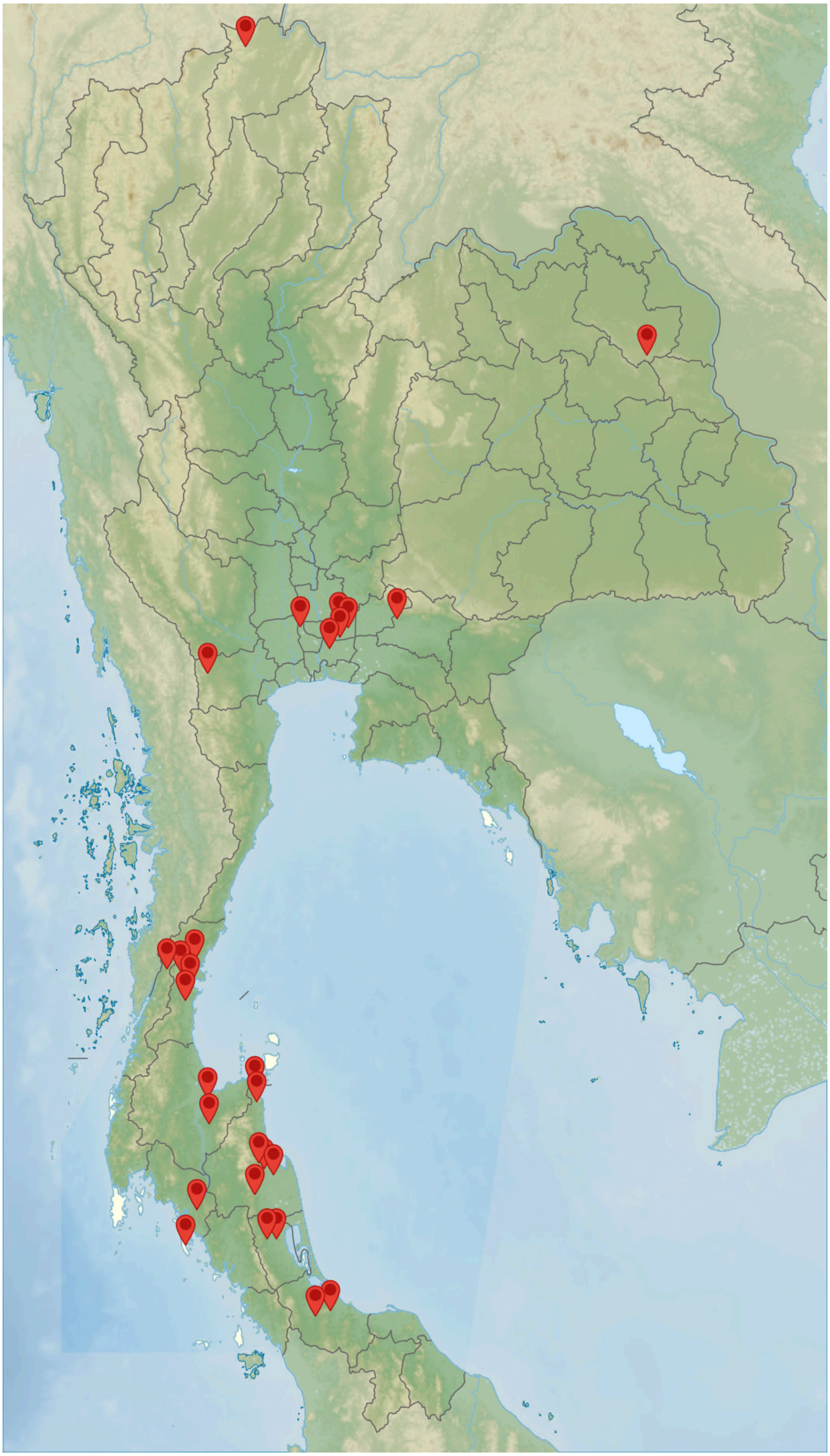

Supplementary Figure S1. A map illustrating the locations of 85 *M. speciosa* accessions in Thailand collected for the population structure study.

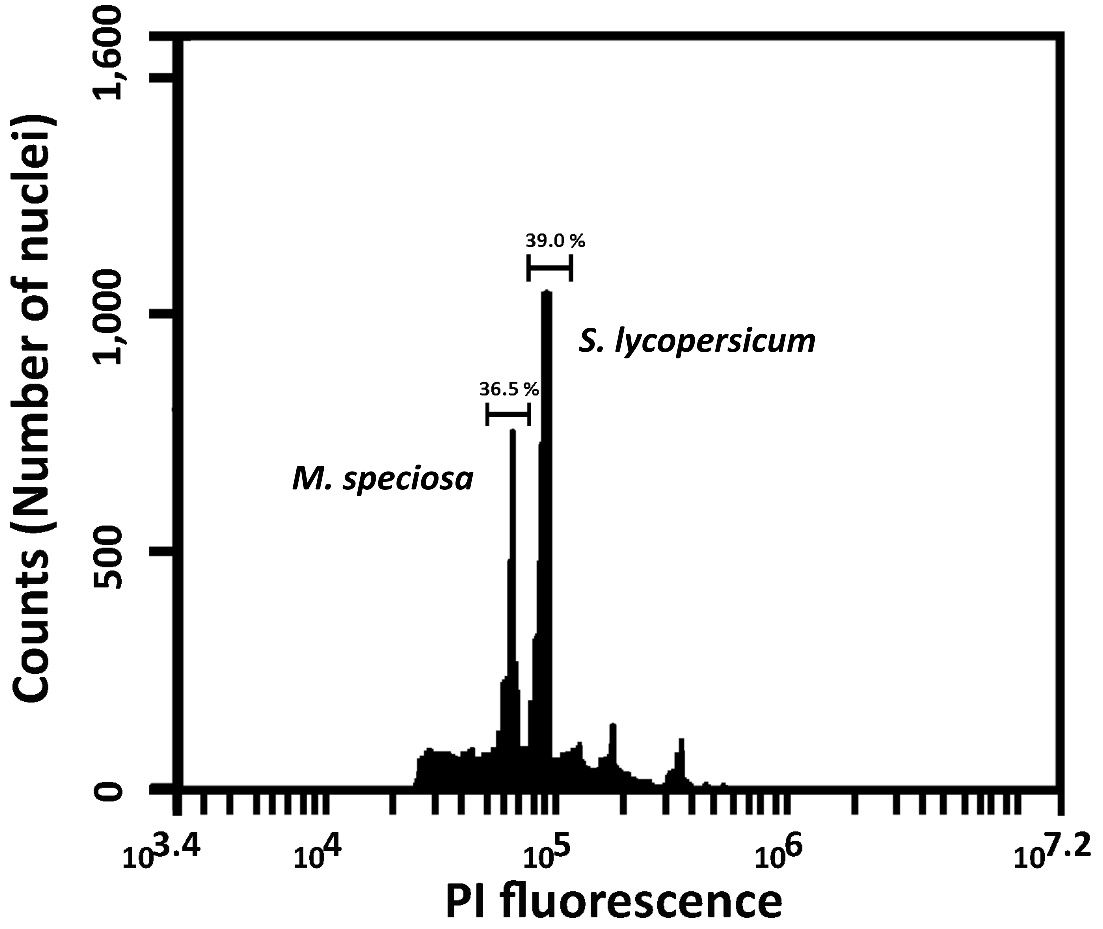

Supplementary Figure S2. Genome size estimation by DNA flow cytometry. Histograms of relative DNA contents obtained after analysis of nuclei isolated from leaf tissues of *M. speciosa* and tomato (*Solanum lycopersicum*; used as a reference standard).

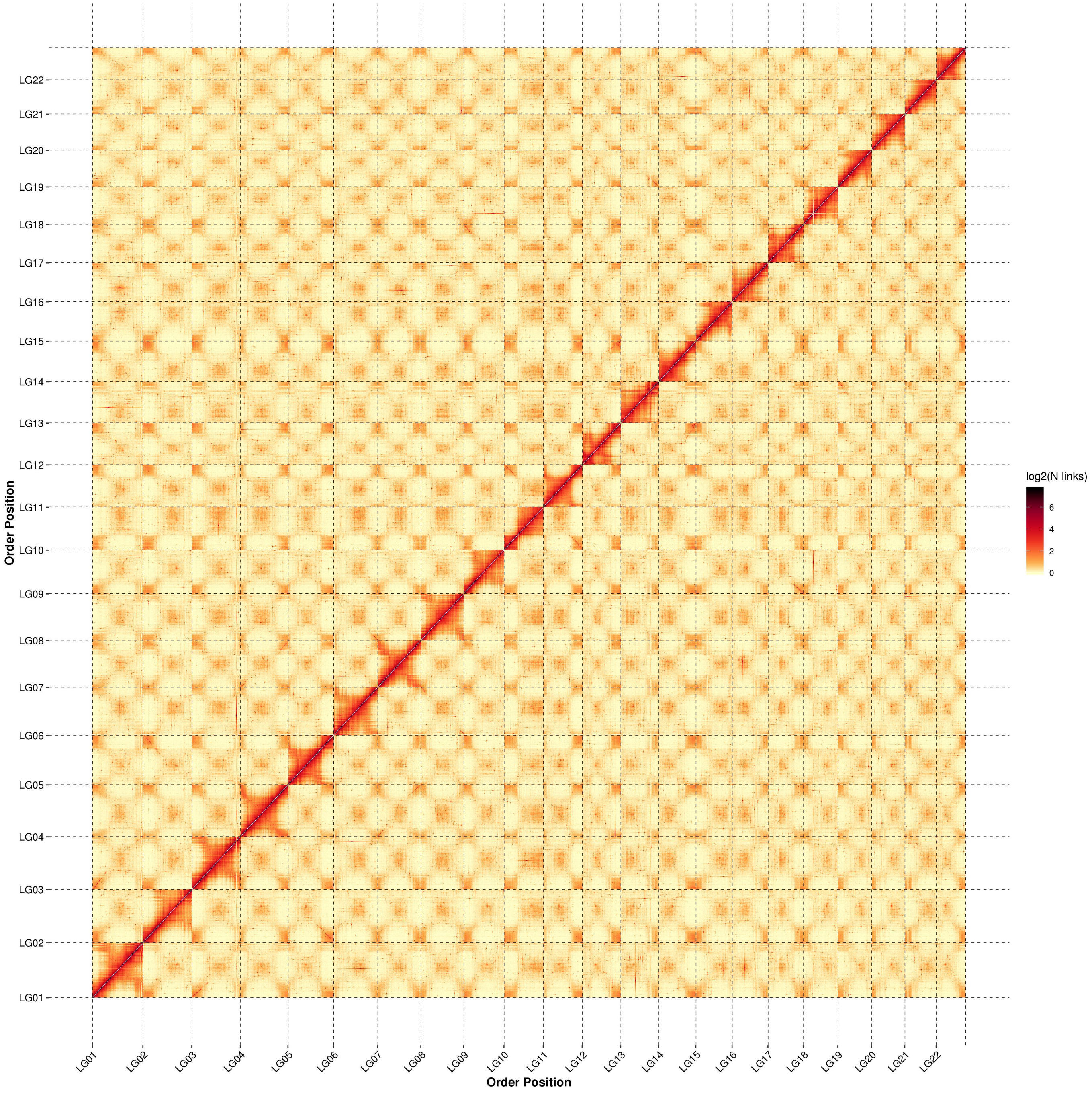

Supplementary Figure S3. Hi-C interaction matrix maps of *M. speciosa* chromosomes. The contact density is indicated by the color scale on the right from dark red (high density) to white (low density).

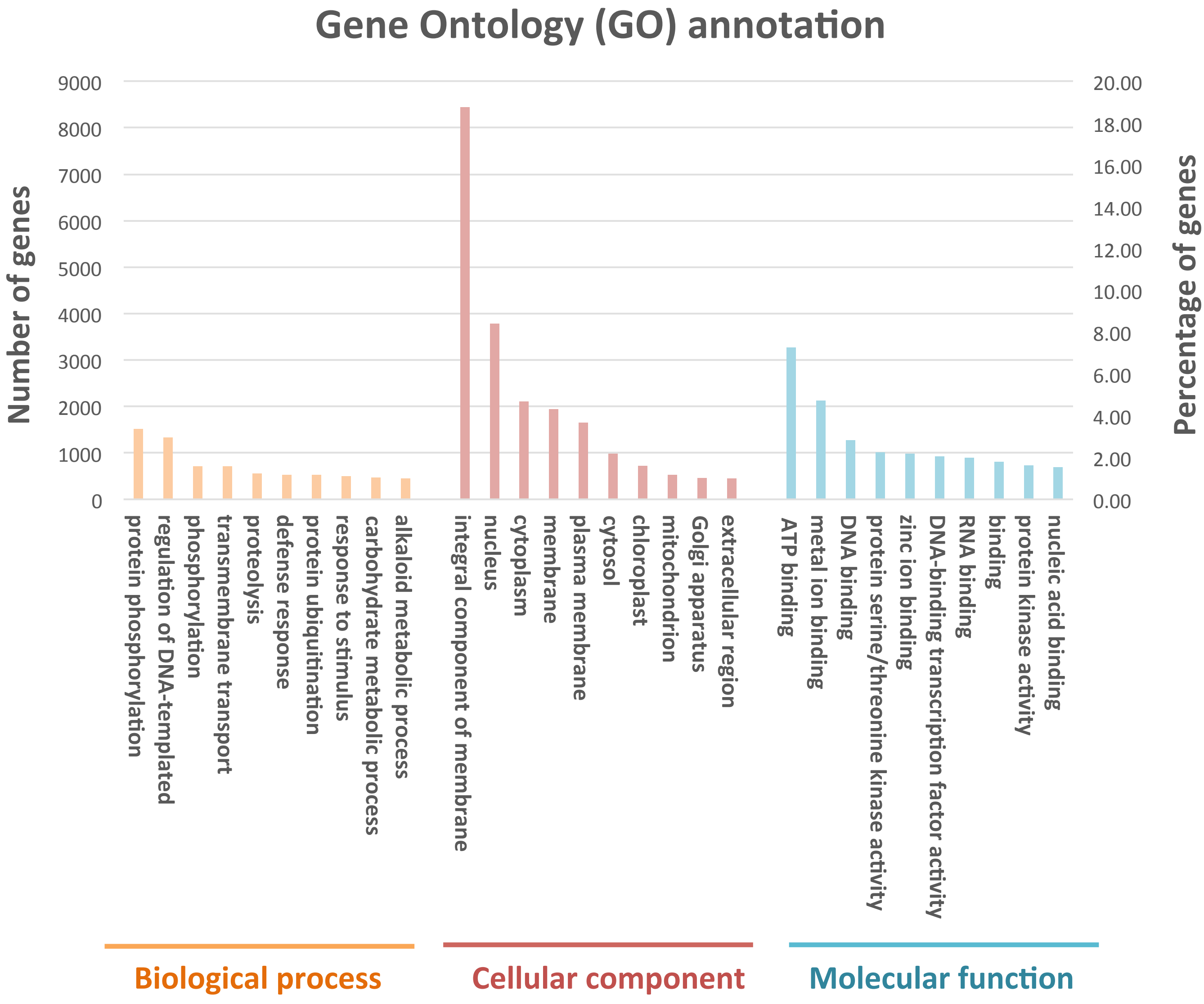

Supplementary Figure S4. Gene Ontology (GO) annotation of *M. speciosa* genes in the assembly.

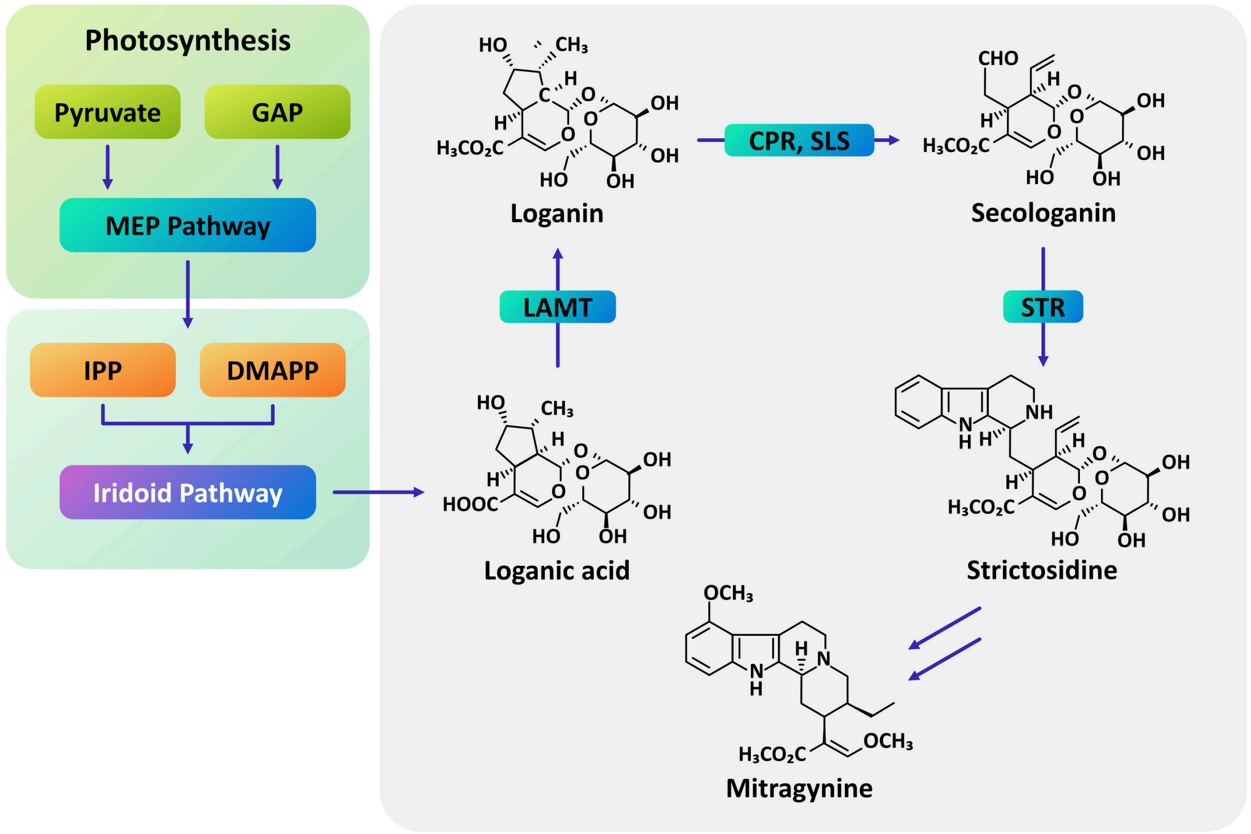

Gene Abbreviation	Gene Name	Number of genes
DXS	1-deoxy-D-xylulose 5-phosphate synthase 2	5
DXR	1-deoxy-D-xylulose-5-phosphate reductoisomerase	1
CMS	4-diphosphocytidyl-methylerythritol 2-phosphate synthase	1
CMK	4-diphosphocytidyl-2-C-methyl-D-erythritol kinase	1
MCS	2C-methyl-D-erythritol 2,4-cyclodiphosphate synthase	1
HDS	GCPE protein	2
HDR	1-hydroxy-2-methyl-butenyl 4-diphosphate reductase	2
GPPS	geranyl pyrophosphate synthase	3
GES	plastid geraniol synthase	3
ISY	iridoid synthase	4
IO	iridoid oxidase	6
LAMT	loganic acid methyltransferase	5
CPR	cytochrome P450-reductase	4
SLS	secologanin synthase/ cytochrome P-450 protein	11
TDC	tryptophan decarboxylase	1
STR	strictosidine synthase	5

Supplementary Figure S5. Mitragynine biosynthesis pathway and the list of genes in the pathway that were identified in the genome assembly.

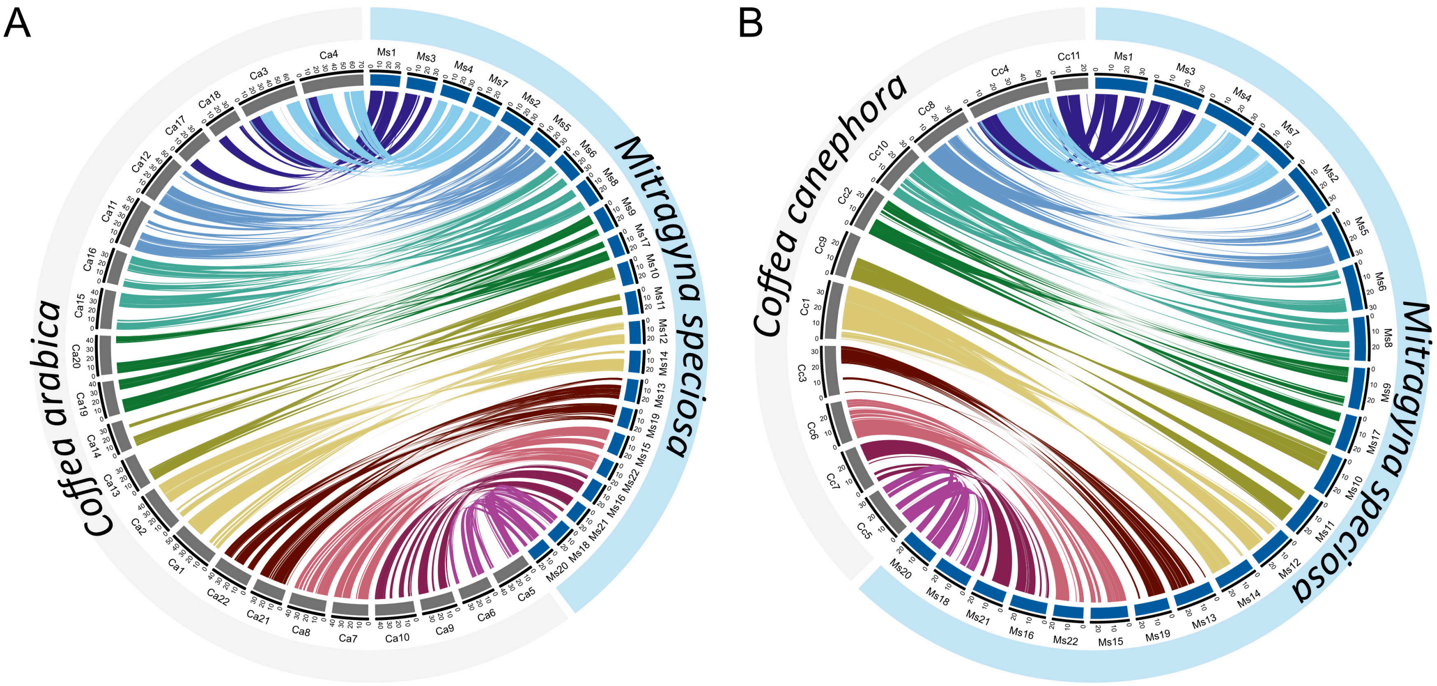

Supplementary Figure S6. Synteny between *M. speciosa* and (A) *C. canephora* (B) *C. arabica*.

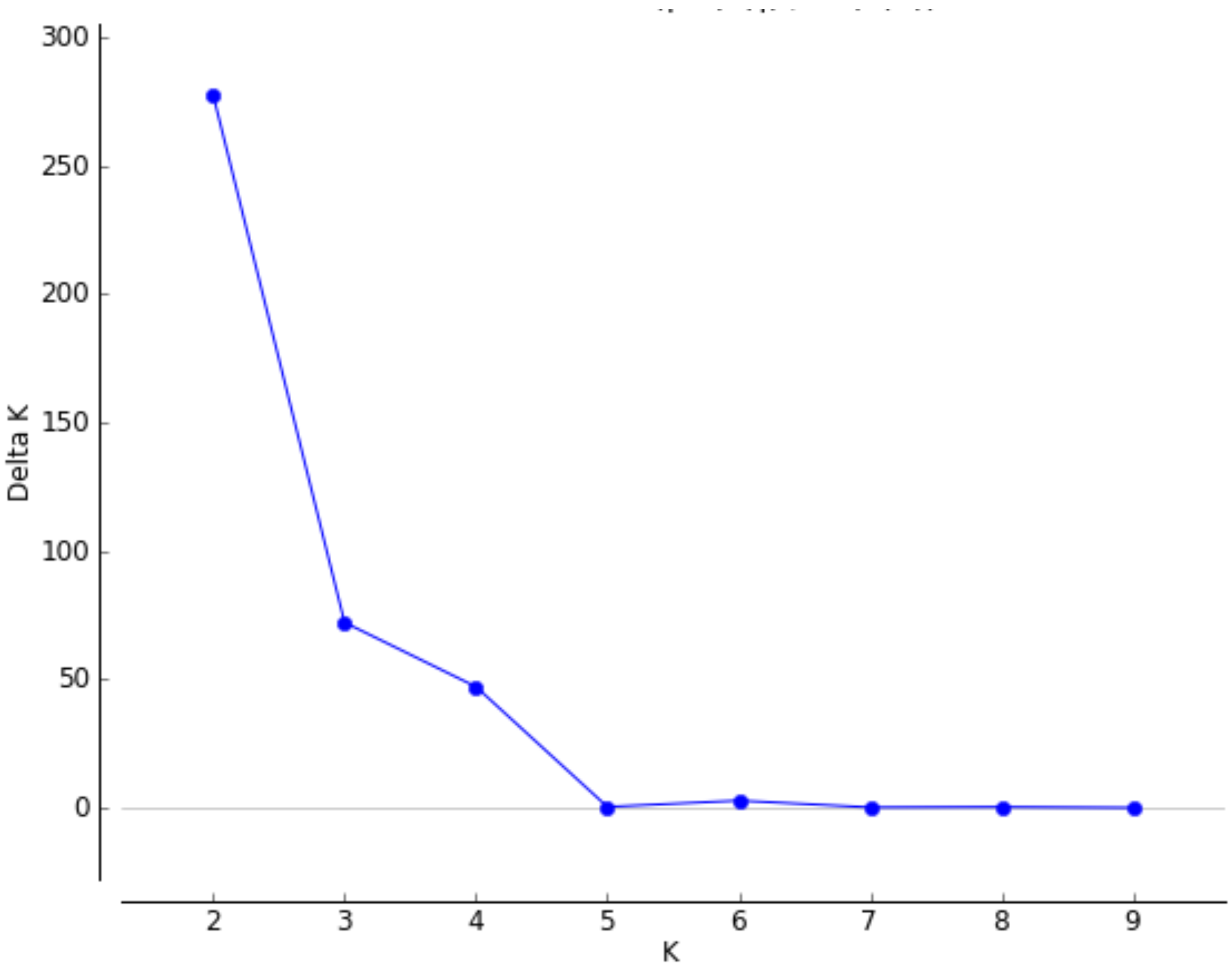

Supplementary Figure S7. Delta *K* values for STRUCTURE analysis of 85 *M. speciosal* accessions. Delta *K* is plotted against the number of modeled gene pools (*K*).

Our assembly: Chromosome 3

Our assembly: Chromosome 11

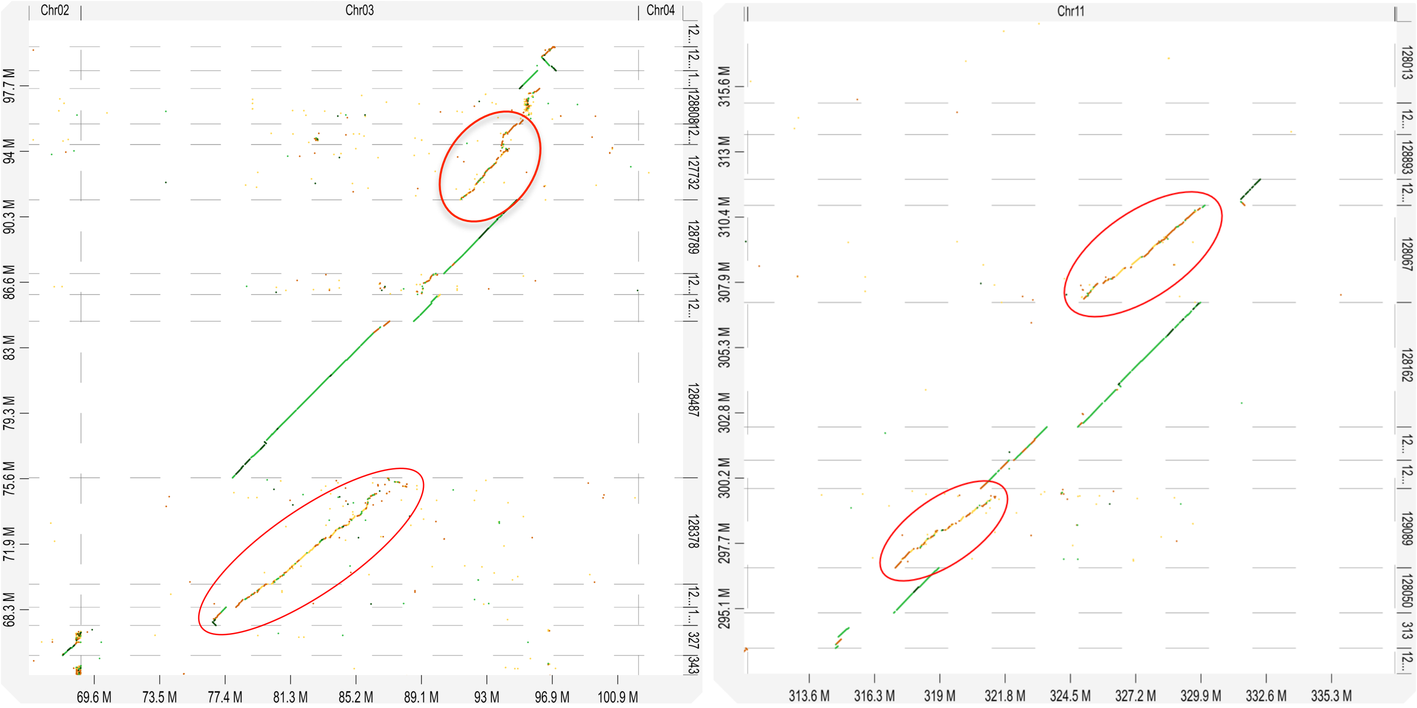

Supplementary Figure S8. Examples of the dot plot alignment of scaffolds/contigs from our genome assembly (X-axis) and the previously published one (Y-axis). Red circles indicated duplicated regions in the assembly published by Brose et al. (2021).
